# Inferring Stabilizing Mutations from Protein Phylogenies: Application to Influenza Hemagglutinin

**DOI:** 10.1371/journal.pcbi.1000349

**Published:** 2009-04-17

**Authors:** Jesse D. Bloom, Matthew J. Glassman

**Affiliations:** Division of Biology, California Institute of Technology, Pasadena, California, United States of America; Harvard University, United States of America

## Abstract

One selection pressure shaping sequence evolution is the requirement that a protein fold with sufficient stability to perform its biological functions. We present a conceptual framework that explains how this requirement causes the probability that a particular amino acid mutation is fixed during evolution to depend on its effect on protein stability. We mathematically formalize this framework to develop a Bayesian approach for inferring the stability effects of individual mutations from homologous protein sequences of known phylogeny. This approach is able to predict published experimentally measured mutational stability effects (ΔΔ*G* values) with an accuracy that exceeds both a state-of-the-art physicochemical modeling program and the sequence-based consensus approach. As a further test, we use our phylogenetic inference approach to predict stabilizing mutations to influenza hemagglutinin. We introduce these mutations into a temperature-sensitive influenza virus with a defect in its hemagglutinin gene and experimentally demonstrate that some of the mutations allow the virus to grow at higher temperatures. Our work therefore describes a powerful new approach for predicting stabilizing mutations that can be successfully applied even to large, complex proteins such as hemagglutinin. This approach also makes a mathematical link between phylogenetics and experimentally measurable protein properties, potentially paving the way for more accurate analyses of molecular evolution.

## Author Summary

Mutating a protein frequently causes a change in its stability. As scientists, we often care about these changes because we would like to engineer a protein's stability or understand how its stability is impacted by a naturally occurring mutation. Evolution also cares about mutational stability changes, because a basic evolutionary requirement is that proteins remain sufficiently stable to perform their biological functions. Our work is based on the idea that it should be possible to use the fact that evolution selects for stability to infer from related proteins the effects of specific mutations. We show that we can indeed use protein evolutionary histories to computationally predict previously measured mutational stability changes more accurately than methods based on either of the two main existing strategies. We then test whether we can predict mutations that increase the stability of hemagglutinin, an influenza protein whose rapid evolution is partly responsible for the ability of this virus to cause yearly epidemics. We experimentally create viruses carrying predicted stabilizing mutations and find that several do in fact improve the virus's ability to grow at higher temperatures. Our computational approach may therefore be of use in understanding the evolution of this medically important virus.

## Introduction

Knowledge of the impact of individual amino acid mutations on a protein's stability is valuable both for understanding the protein's natural evolution and for altering its properties for engineering purposes. Experimentally measuring the effects of mutations on protein stability is a laborious process, so a variety of methods have been devised to predict these effects computationally. Most existing methods rely on some type of physicochemical modeling of the mutation in the context of the protein's three-dimensional structure, often augmented by information gleaned from statistical analyses of protein sequences and structures. These types of methods are moderately accurate at predicting the effects of mutations on the stabilities of small soluble proteins [1]–[8]. There is little or no published data evaluating their performance on the larger and more complex proteins that are frequently of greatest biological interest, although it might be expected to be worse given the greater difficulty of modeling larger structures.

An alternative approach to predicting the effects of mutations on protein stability utilizes the information contained in alignments of evolutionarily related sequences. This approach, which was originally introduced by Steipe and coworkers [9], envisions an alignment of related sequences as representing a random sample of all possible sequences that fold into a given protein structure. Based on a loose analogy with statistical physics, the frequency of a given residue in the sequence alignment is assumed to be an exponential function of its contribution to the protein's stability (just as the Boltzmann factor in statistical physics relates the probability of a microscopic state to the exponential of its energy). This is often called the “consensus” approach, since it always predicts that the most stabilizing mutation will be to the most commonly occurring (consensus) residue. The consensus approach has proven to be surprisingly successful, with a wide range of studies supporting the basic notion that stabilizing residues tend to appear more frequently in sequence alignments of homologous proteins [10]–[17].

But although it is often effective, the consensus approach suffers from an obvious conceptual flaw: alignments of natural proteins do not represent random samples of all possible sequences encoding a given structure, but instead are highly biased by evolutionary relationships. A particular residue might occur frequently because it has arisen repeatedly through independent amino acid substitutions, or it might occur frequently simply because it occurred in the common ancestor of many related sequences in the alignment. The sequence evolution of even distantly related protein homologs is non-ergodic (as evidenced by the fact that sequence divergence continues to increase with elapsed evolutionary time), and so this problem will plague all natural sequence alignments. Therefore, it would clearly be desirable to extract information about protein stability from sequence alignments using a method that accounts for evolutionary relationships.

In fact, there are already highly developed mathematical descriptions of the divergence of evolving protein sequences. The widely used likelihood-based methods for inferring protein phylogenies employ explicit models of amino acid substitution to assess the likelihood of phylogenetic trees [18]. However, these methods make no effort to determine how selection for protein stability might manifest itself in the ultimate frequencies of amino acids in an alignment of evolved sequences. Instead, in their simplest form, these phylogenetic methods simply assume that there is a universal “average” tendency for one particular amino acid to be substituted with another (these “average” substitution tendencies are typically given by PAM, BLOSUM, or JTT matrices). More advanced phylogenetic methods sometimes allow for different “average” substitution tendencies for different classes of protein residues (such as surface versus core residues, or residues involved in different types of secondary structures) [19]–[24]. Still other methods use simulations or other structure-based methods to derive site-specific substitution matrices for different positions in a protein [25]–[28]. However, none of these methods relate the substitution probabilities to the effects of mutations on experimentally measurable properties such as protein stability, nor do they provide a method for predicting the effects of the mutations from the protein phylogenies.

Here we present an approach for using protein phylogenies to infer the effects of amino acid mutations on protein stability. We begin by describing a conceptual framework that quantitatively links a mutation's effect on protein stability to the probability that it will be fixed by evolution. We then show how this framework can be used to calculate the likelihood of specific phylogenetic relationships given the stability effects of all possible amino acid mutations to a protein. Our actual goal is to do the reverse, and infer the stability effects given a known protein phylogeny. To robustly accomplish this, we use Bayesian inference with informative priors derived from an established physicochemical modeling program. We compare the inferred stability effects to published experimental values for several proteins, and show that our method outperforms both the physicochemical modeling program and the consensus approach. Finally, we use our method to predict mutations that increase the temperature-stability of influenza hemagglutinin, a complex multimeric membrane-bound glycoprotein for which (to our knowledge) stabilizing mutations have never previously been successfully predicted by any approach. We introduce the predicted stabilizing mutations into hemagglutinin, and experimentally demonstrate that several of them increase the temperature-stability of the protein in the context of live influenza virus. Overall, our work presents a unified framework for incorporating protein stability into phylogenetic analyses, as well as demonstrating a powerful new approach for predicting stabilizing mutations.

## Results

### A framework relating the biophysical impact of amino acid mutations to the frequency with which they are fixed during neutral evolution

We begin by introducing a conceptual framework that relates the probability that a specific amino acid mutation will be selectively neutral (and so have an opportunity to spread by genetic drift) to its effect on protein stability. Because this conceptual framework forms the starting point for subsequent mathematical inference, it is necessarily highly simplified. It is based on several assumptions which, although motivated by biophysical considerations, are subject to many exceptions. Below we outline these assumptions, and mention some of the exceptions. We hope the reader will become convinced that this conceptual framework strikes a reasonable balance between being realistic and mathematically tractable. The conceptual framework that we describe has previously been successfully employed in simulations [29],[30], and later in theoretical treatments [31],[32], of protein evolution.

**Figure 1:**
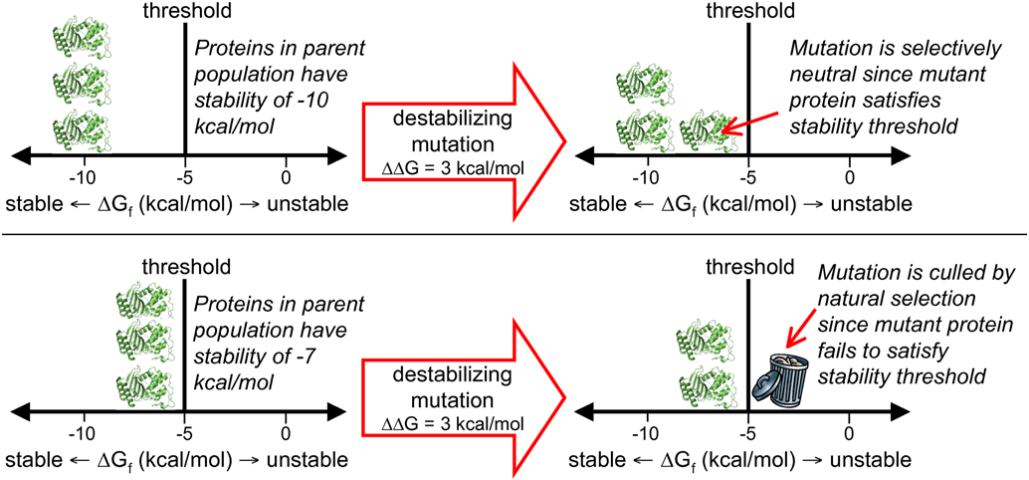
A stability threshold model of protein evolution. Proteins are assumed to be functional if and only if they are more stable than some minimal threshold (in the figure, 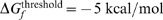, which is a typical value for natural proteins [53]; note that more stable proteins have more negative 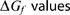 values). When a particular destabilizing mutation (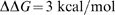) occurs, the evolutionary result will depend on the stability of the proteins in the parent population. When the parent proteins are sufficiently stable (top panel), the mutant protein still satisfies the threshold, and so the mutation has the opportunity to spread by neutral genetic drift. But when the parent proteins are not sufficiently stable (bottom panel), the mutant protein fails to stably fold, and is eliminated by natural selection. Therefore, the probability that a mutation that induces a stability change of 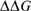 will have an opportunity to spread by neutral genetic drift is simply the probability that the parent protein has a stability 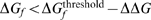.

We assume that evolution selects only for a protein's biochemical function, and is indifferent to its precise stability provided that the protein folds with sufficient stability to perform its function. This assumption is imperfect, since some proteins are natively unfolded [33], only kinetically stable [34], or specifically selected for marginal stability in order to aid in regulation [35]. In addition, mildly destabilized proteins might retain partial function while being subject to weak negative selection. This assumption nonetheless captures the overriding idea that most proteins have evolved to fold to stable structures in order to perform biochemical functions that are the actual dominant targets of natural selection. With this assumption, proteins can be viewed as having to satisfy a minimal stability threshold in order to avoid being culled by natural selection (see Figure 1).

We further assume that all protein mutants that satisfy the stability threshold are equally functional, while all mutants that fail to satisfy the threshold are nonfunctional. This assumption has the mathematically desirable property that it neatly divides all mutants into one of two categories (sufficiently stable or nonfunctional). Of course, we recognize that this assumption is not strictly true, since one could fill many pages documenting mutations that are deleterious despite preserving stability. For example, mutations can specifically interfere with a protein's function (such as altering an enzyme's activity)—but experiments have shown that such mutations are rare compared to the much larger number that affect stability [36]–[39]. Mutations can also be deleterious if they increase a protein's propensity to aggregate [40]–[43] or interfere with its folding [44] or unfolding [16],[45] kinetics—but quantifying a mutation's impact on stability provides a partial proxy for these effects since aggregation propensity [40], folding rate [46]–[49], and kinetic stability [16] are correlated with stability. Mutations can also have other deleterious effects, such as altering mRNA stability [50], codon usage [51], or the accuracy and efficiency of translation [43],[51],[52]. We mention these myriad exceptions to explicitly acknowledge their existence. Nonetheless, from here forward we will use the concept of stability threshold selection to develop a mathematical relationship between protein stability and evolution.

Figure 1 illustrates the stability threshold view of evolution that we have just described. In this figure, a protein's stability is quantified by its free energy of folding (its 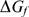), and the effects of mutations by the change they induce in the free energy of folding (their 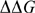 values) [53]. For proteins that do not fold reversibly, some alternative experimental measure of stability (such as resistance to thermal denaturation [54] or proteolysis [55]) is clearly required, but the concept remains the same. The key implication of Figure 1 is that the evolutionary impact of a mutation can depend on the stability of the parent protein into which it is introduced, with a moderately destabilizing mutation being neutral in the context of a stable parent but lethal to a marginally stable parent. That mutational tolerance is indeed enhanced by extra stability in this fashion has been experimentally verified for several proteins [56]–[58]. This idea provides a basis for forsaking the traditional approach of using pre-specificied “average” amino acid substitution matrices, and instead adopting the view that the frequency of a particular substitution tells us something about its impact on protein stability. Much of the rest of this paper deals with the mathematical mechanics of how to use the substitution frequencies implied by a set of protein homologs to infer the effects of individual mutations on stability.

### Sequence evolution without any selection

To introduce the mathematical analysis, begin by considering protein sequence evolution in the absence of any selection on amino acid composition. Even in the absence of selection, some amino acid substitutions are more likely than others due to the structure of the genetic code and unequal frequencies of different types of nucleotide mutations. In order to express the probabilities of various types of mutations only in terms of amino acid identities, assume that the distribution of codons encoding each amino acid is always at equilibrium. For example, assume that all glycines at all times have the same probability of being encoded by the GGG codon. With this assumption, the current state of a residue can be described by its amino acid identity rather than its codon identity (see [59] for an evolutionary model that operates at the codon-level). Given that a particular position is currently amino acid 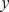, let 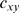 denote the probability that a single nucleotide mutation to the codon at this position changes the identity to amino acid 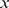. Nonsense mutations (to stop codons) are assumed to be immediately eliminated by selection, and so leave the codon unchanged. All other mutations are assumed to be neutral. Therefore, all nonsense and synonymous mutations contribute to 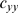, and all nonsynonymous mutations contribute to 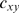 with 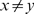. Denote the set of all 20×20 = 400 values of 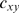 as 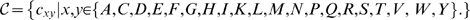. Note that 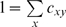, since each mutation either leads to a new amino acid (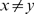) or leaves the amino acid unchanged (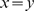).

Let 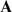 be the 20×20 matrix with off-diagonal elements 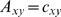 and diagonal elements 
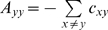
. Let 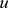 be the rate at which an individual codon experiences a nucleotide mutation, so that each codon experiences an average of 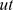 mutations after an elapsed time of 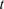. It is assumed that all codons in the protein experience the same mutation rate 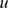. As will be seen below, the full model still allows variation in the rate at which substitutions accumulate at different residues, but this variation is caused by selection for stability rather than by differences in the underlying rate of mutation. Without selection for stability, the probability that a residue that is initially 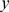 will be 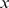 after an elapsed time of 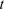 is given by the element 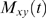 of the matrix 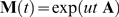 [18]. After a sufficiently long period of time (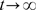), the probability to find some specific amino acid 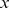 is the same across all positions of the protein, and is given by element 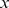 of the right eigenvector of 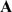 corresponding to the unique zero eigenvalue (the uniqueness of this eigenvector is guaranteed by the Perron-Frobenius theorems, since 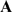 plus the identity matrix will be an irreducible and acyclic stochastic matrix). Of course, real proteins tend to prefer some amino acids at certain positions, such as hydrophobic residues in the core. The substitution model that has just been described fails to account for these preferences. The next section explains how this problem can be remedied by incorporating selection for stability.

### Substitution probabilities in the presence of selection for stability

The situation described in the previous section changes fundamentally in the presence of selection for protein stability, since mutations will be eliminated if they destabilize a protein beyond the threshold. Specifically, let 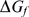 be the stability (free energy of folding [53]) of the parent protein and let 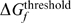 be the minimal stability required by the threshold, so that the protein has extra stability 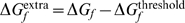. Only those mutations that leave 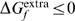 have a chance to be fixed by evolution (more negative 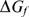 values indicate more stable proteins). Let 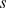 be the sequence of a protein of the length 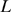, and let 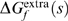 be the extra stability of this protein. Mutating residue 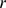 of the protein from its current identity 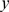 to some new amino acid 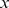 induces a stability change of 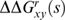. Under the stability threshold model, this mutation can become fixed if and only if 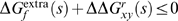.

This description, in which 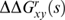 for mutating residue 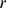 is a function of the parent sequence 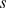 as well as the residue identities 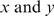, is completely general. However, it is not useful. The reason for this lack of utility is that there are 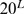 different possible protein sequences 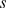, since each of the 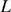 positions in the protein can take on any of the 20 amino acids. Since 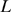 for a typical protein is several hundred residues, the number of different 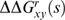 values exceeds the number of atoms in the Universe. This many values cannot reasonably be specified *a priori* or inferred from available sequence data.

However, the situation can be made more tractable by assuming that 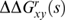 is independent of 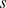, and so is equal to the same value of 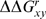 for all sequences. This assumption is equivalent to saying that the 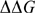 values for mutations to different residues are independent and additive, which implies that the 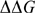 value of a mutation does not depend on the sequence background in which it appears. This assumption is clearly not completely true, since protein stability depends on cooperative interactions among many residues. However, empirically it appears that the assumption of independent and additive 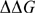 values is nonetheless actually rather good. For example, a number of biochemical studies have indicated that the 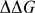 values for a modest number of amino acid mutations are frequently independent and additive [60]–[65]. Of particular relevance is a study by Fersht and Serrano [65] of the amino acid substitutions separating the homologous proteins binase and barnase, which have 85% sequence identity. They found that combinations of these substitutions had additive effects on stability, indicating that the 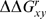 values are very nearly constant among the sequences that occurred during the evolutionary divergence of these two proteins. This high degree of independence and additivity of experimentally measured 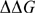 values may be due to the fact that pairwise amino-acid interaction potentials can be accurately approximated by independent sites [28],[66]. Regardless of the underlying reasons, at least at modest levels of sequence divergence, there is experimental evidence that the approximation of constant 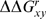 values is quite accurate.

Assuming that 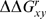 is independent of the particular sequence background greatly reduces the number of these values that need to be determined. To count the number of unique 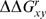 values, note that any closed loop in the space of protein sequences yields no net change in stability. That is, 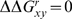 (since there is no stability change when there is no mutation), 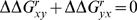 (since mutating 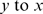 and then back to 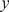 does not change the sequence), and 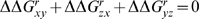 (since this combination of mutations leaves the sequence unchanged). Therefore, all 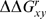 values can be determined with reference to mutating an arbitrarily chosen amino acid, which is here taken to be alanine (A). There are 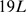 different 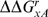 values, since each of the 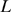 residues can be mutated to any of the 19 non-alanine amino acids. The specification of all 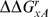 values allows any 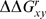 value to be calculated as
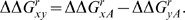
(1)


All 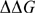 values are therefore uniquely determined by the set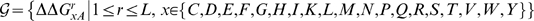 of 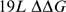 values. This paper will show that the elements of 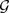 can be reasonably inferred using informative Bayesian priors.

First, assume that 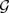 is known and consider the problem of using this knowledge to determine whether selection will tolerate a particular mutation to some specified protein sequence. Let 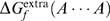 be the extra stability of a sequence composed entirely of the alanine reference amino acid. The extra stability of any protein sequence 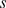 can be calculated from 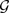 and 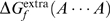 as
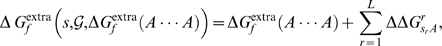
(2)where 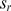 is the amino acid at residue 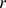 of sequence 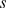. Under the stability threshold model, mutating residue 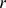 of the folded protein with sequence 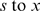 is acceptable to selection if and only if 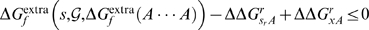. It may be possible to use this formulation to develop a mathematically tractable description of protein evolution. However, the situation is complicated by the fact that the acceptability of a mutation depends on the protein sequence 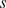. Therefore, describing protein evolution using Equation 2 requires estimating the stability of each sequence that occurs along the phylogenetic tree, and averaging over all possible sequence paths. This paper circumvents this difficult task by making the additional (mean-field) approximation that the acceptability of a specific mutation depends on the average distribution of 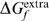, rather than on the exact stability of the protein sequence in which the mutation occurs. In other words, we take the probability that mutating residue 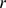 from 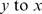 is neutral to be equal to the probability that 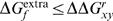. This mean-field approximation eliminates all coupling between substitutions at different sites in the protein.

**Figure 2:**
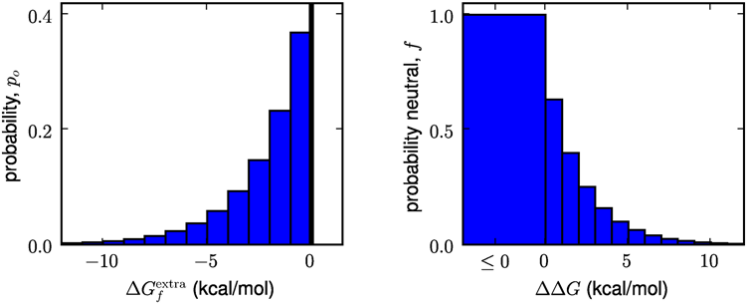
Stability distributions and fixation probabilities. The panel at left show the probability 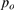 that a protein in an evolving population will have extra stability 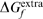, as given by Equation 3. The panel at right shows the probability 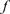 that a mutation that causes a stability change of 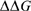 will be neutral, as given by Equation 4. The units for 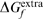 are arbitrary; for concreteness here we give them units of kcal/mol.

With this mean-field approximation, the issue becomes determining the average distribution of stabilities in an evolving population of proteins. This problem has been treated previously by simulations [29] and mathematically through matrix [31] and diffusion [32] equation approaches. The average distribution of stabilities turns out to depend on the degree of polymorphism in the population, with highly polymorphic populations (those with the product of the population size 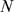 and the per sequence per generation mutation rate 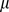 much greater than one) evolving to greater average stabilities than populations that are mostly monomorphic (those with 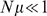) [31],[67],[68]. Here we will consider only the case where the population is mostly monomorphic, so that all proteins tend to have converged to the same stability before a new mutation occurs (as is the case for the proteins shown in Figure 1). This choice is dictated by the fact that we are unclear how to incorporate the secondary selection for mutational robustness that occurs in highly polymorphic populations [31],[67],[68]. We acknowledge that some of the proteins that we analyze later in this paper (particularly influenza hemagglutinin) may actually evolve in populations that are highly polymorphic, and suggest that a mathematical treatment recognizing this fact is an area for future research. Given our choice to consider only the case where the population is mostly monomorphic, we will adopt the mathematical formalism described in [31] for the limit when 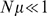 (the more compact diffusion-equation approach of Shakhnovich and coworkers [32] cannot be used since it only applies when 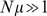). Following [31], we discretize the continuous variable of extra protein stability 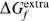 into small bins of width 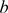, and assign a protein to bin 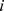 if it has extra stability such that 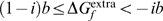, where 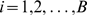. Here 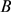 is some large integer giving an upper limit on the number of stability bins (so that all proteins in the evolving population have 
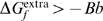
). Note that all folded proteins fall into one of these bins, since proteins with 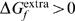 fail to fold under the stability threshold model. Reference [31] finds that the distribution of average protein stabilities is well approximated by an exponential (see the middle panels of Figure 2 of this reference, or alternatively Figure 2A of [29]), such that the probability 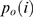 that a protein in the evolving population has extra stability that falls in bin 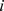 is
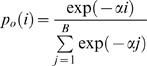
(3)where 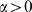 is a constant describing the steepness of the exponential. Figure 2 shows this distribution of protein stabilities graphically. Note that this exact mathematical form for 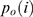 is not proven in [31], but simply that all numerical solutions give distributions for 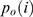 that resemble this form. Other mathematical forms could be chosen for 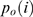 without altering the mathematical analysis that follows, although they might affect the actual numerical values that are ultimately inferred for the 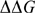 values. In particular, in highly polymorphic populations, the distribution of stabilities is peaked at a value slightly below the stability threshold (see right panels of Figure 2 of [31], Figure 2 of [32], or Figure 2B of [29]) rather than being an exponential. However, any distribution in which highly stable proteins are rare and marginally stable proteins are common should lead to qualitatively similar inferred 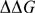 values, since the subsequent analysis only employs the cumulative distribution function of 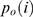 in a rather coarse manner. Given the definition of 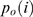 in Equation 3, the exact numerical for 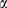 simply sets a scale for the 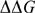 values (in conjunction with the bin size 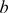, it determines their units). As is described later in this paper, in our actual computational implementation, we chose a value for 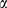 that placed the magnitude of the inferred 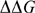 values in the same dynamic range as the informative priors.

Using the mean-field approximation for 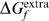, the probability that a mutation is neutral can now be computed from 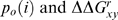. Stabilizing mutations are always neutral, while destabilizing mutations are neutral with a probability equal to the fraction of time they will not unfold a protein with extra stability drawn from 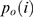. Mathematically, the probability 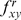 that mutating residue 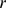 from 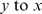 is neutral is
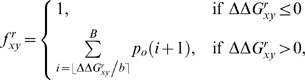
(4)where 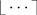 is the nearest integer function. Figure 2 graphically illustrates the probability that a mutation will be neutral given its 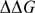 value. Define 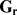 to be the matrix with off-diagonal elements 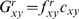 and diagonal elements 
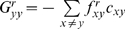
. The probability that a substitution changes position 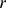 of the protein from its original identity of amino acid 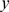 to amino acid 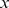 after an elapsed time 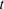 is therefore given by element 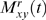 of the matrix 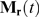 defined by
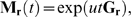
(5)where 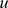 is the per codon mutation rate as defined above. The previous section showed that in the absence of selection for stability, the probability of finding some specific amino acid at a position was equal for all positions in the limit of long time. With selection for stability, this is no longer the case. Let the probability 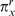 of finding residue 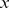 at position 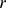 in the long-time limit be given by element 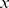 of the vector 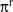. The vector 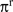 represents the stationary solution to Equation 5, and so is the probability vector (entries sum to one) that satisfies the eigenvector equation
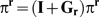
(6)where 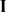 is the identity matrix. Given a value of 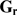, the uniqueness of 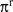 is guaranteed by the Perron-Frobenius theorems, since 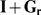 is a nonnegative and acyclic stochastic matrix. Since 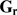 depends on the 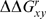 values for the stability effects of mutations, the probabilities of observing amino acids at specific positions in the sequence depends on their stability contributions.

### Bayesian framework for inferring 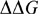 values from sequence data

The previous section describes how the probabilities of specific substitutions to an evolving protein are shaped by the set 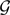 of 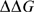 values. In practice, we simply have some set 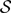 of homologous protein sequences. The inference problem is how to estimate 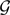 from 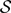. In so doing, we will also need to estimate 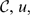, and the phylogenetic relationship among the sequences. The approach we will take is to use Bayesian inference [18], [69]–[71] to estimate 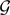 from 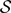. Sadly, the approach is not fully Bayesian, since computational limitations require some important quantities to be estimated by alternative means. Hopefully in the future, the computation can be recast in fully Bayesian terms.

**Figure 3:**
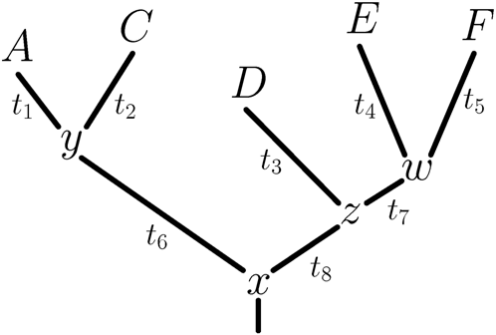
An example phylogenetic tree 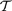. This tree shows the sequence data 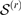 for five sequences at a single site 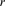. The amino acid codes at the tips of the branches (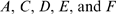) show the residue identities for the five sequences at this site. The variables at the internal nodes (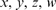) are the amino acid identities at the site for the ancestral sequences, and must be inferred. The branch lengths (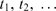) are proportional to the time since the divergence of the sequences.

The inference problem begins with the set 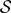 of homologous protein sequences. Here it is assumed that these proteins have diverged from a common ancestor by point mutations (any insertions/deletions are ignored), and that there is no recombination within the protein coding sequences. It is further assumed that all of the homologous sequences can be aligned in a fashion that puts their residues in a one-to-one correspondence. In mathematical terms, 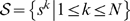 consists of 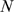 homologous sequences of length 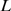, with 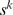 denoting the 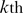
th sequence. For each sequence 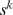, we know the identity 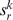 of the amino acid at position 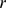 (where 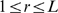). The set of amino acid identities for all 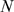 proteins at a single site 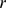 is denoted by 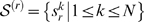. The evolutionary relationship among the sequences is given by some phylogenetic tree 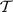. Here 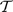 is taken to specify both the topology and branch lengths of a rooted phylogenetic tree, as shown in Figure 3.

Using the prescription of the previous section to calculate the substitution probabilities, it is possible to calculate the likelihood 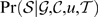 of observing some set of sequences given the 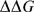 values. Here we briefly outline how this calculation would proceed, closely paralleling the description by Felsenstein [18] of the pruning-based likelihood calculation method he developed [72],[73]. Making the standard phylogenetic assumption that evolution at each site is independent,
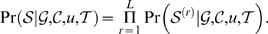
(7)


Consider the computation for some specific site 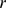. Figure 3 shows the phylogenetic tree 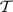 giving the evolutionary relationship among 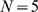 sequences, and the sequence data 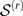 for site 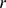 of these sequences. Given this tree in Figure 3, the likelihood for site 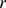 is computed by summing over the twenty possible amino acid identities at each internal node,
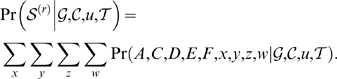
(8)


Assuming the lineages are independent, the probabilities on the right side of Equation 8 can be decomposed as a product,
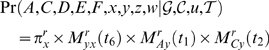


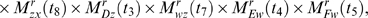
(9)where the 
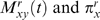
 values are calculated from 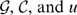 using Equations 5 and 6. Note that Equation 9 assumes that the sequences have evolved for a sufficiently long period of time that the probability of observing residue 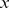 at position 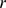 at the root of the tree is the long-time limit 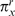. Using the pruning approach of Felsenstein, Equations 8 and 9 can be combined to yield
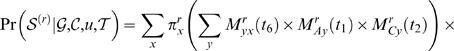


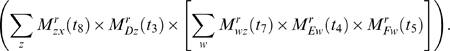
(10)


Equations 7 and 10 provide a method for computing 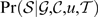. But goal of this analysis is to infer the 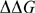 values from the sequences, which is equivalent to computing 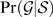. Using Bayes' Theorem,
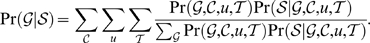
(11)


Solving this equation would yield a fully Bayesian inference of 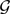 by summing over the unknown variables 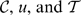. In principle, this equation could also be used to estimate another phylogenetic variable (such as 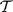) by swapping this variable with 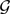 in the equation.

However, in practice, the approach taken here will not be the fully Bayesian estimate given by Equation 11. Instead, to reduce the variable sampling space, other methods will be used to make single-value estimates for each of 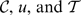, so that
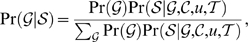
(12)where 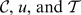 have been assigned fixed values. Given a prior 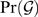 over the 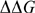 values, the right-hand side of Equation 12 can be estimated numerically. One attractive aspect of this approach is that there is a basis for specifying a meaningful prior over 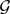, since 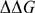 values can be measured experimentally [53],[74], or more easily predicted with at least mild accuracy by one of the available physicochemical modeling programs [1]–[8]. Equation 12 can in principle be solved by Markov chain Monte Carlo (MCMC) methods [69]–[71] to yield a full estimate of the probability distribution 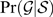. But we are interested in obtaining estimates for the individual 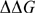 values contained in 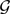, since it is these values that have physical meaning. Therefore, we take the 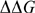 values of the maximum *a posteriori* value 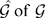, defined as
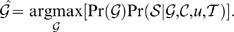
(13)


In the next section, we describe the specific computational approach we have used to solve Equation 13 to obtain the 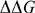 values from an alignment of homologous protein sequences.

### Implementation of a computational approach for inferring 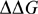 values from sequence data

In this section, we describe the computer program we have developed to infer 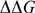 values from the sequences of protein homologs by solving Equation 13. Solving this equation requires specification of the phylogenetic tree 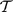, the underlying amino acid mutation probabilities 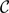, the mutation rate 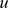, and a prior distribution 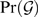 over the 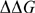 values. Solving the equation also requires a numerical method for maximizing the argument of the 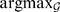 function. We implemented our strategy using the Python programming language, and termed the resulting program PIPS (**P**hylogenetic **I**nference of **P**rotein **S**tability). This program was used to analyze cold shock protein, ribonuclease HI, thioredoxin, and H1 influenza hemagglutinin as described below. The PIPS source code and the full raw data from the analyses in this paper are available at http://openwetware.org/wiki/User:Jesse_Bloom.

We built the phylogenetic tree 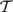 from the set 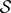 of homologous protein sequences using the PHYLIP package [75]. The protein sequences of the homologs were aligned using ProbCons [76] (for cold shock protein, ribonuclease HI, and thioredoxin) or MUSCLE [77] (for influenza hemagglutinin). Phylogenetic trees of these aligned protein sequences were then constructed using the distance-based method of PHYLIP's “neighbor” program. For cold shock protein, ribonuclease HI, and thioredoxin, the trees were built using the UPGMA method to create rooted trees that conformed to the assumption of a molecular clock. For influenza hemagglutinin, the variation in the date of isolation of the sequences is substantial relative to their divergence, so the neighbor-joining method (no molecular clock) was used to construct a tree which was rooted to an outgroup sequence.

We calculated the underlying amino acid mutation probabilities 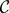 under the assumption that each amino acid was equally likely to be encoded by any of its codons. The probability 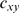 that a single mutation changed amino acid 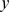 to 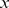 was the probability that a random nucleotide mutation to one of the codons for 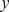 yielded a codon for 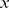, averaged over all of the codons for 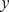. There is evidence that the transition-to-transversion ratio for influenza evolving in humans is somewhere in the range of five [78], so for hemagglutinin we assumed that the nucleotide mutations were made with this bias. We are aware of no clear evidence about the transition-to-transversion ratio for cold shock protein, thioredoxin, and ribonuclease HI, so for these proteins we assumed a ratio of 0.5, which is the expectation in the absence of any mutational bias [79]. We recognize that more accurate amino acid mutation probabilities are likely to be derived from a codon-based model [59], and suggest that incorporating such a model is an area for future work.

The mutation rate 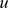 represents the number of nucleotide mutations to a codon that occur for each substitution that is fixed along the branches of the phylogenetic tree (branch lengths are measured in amino acid substitutions per site). Since our program is not yet sufficiently advanced to co-estimate 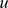 from the sequence data, we had no strong rationale for assigning a particular value to 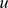. We chose a value of 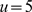, which corresponds to 20% of nucleotide mutations leading to a tolerated amino acid mutation. While we cannot provide an independent justification for this choice of 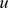, the inferred 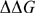 values were fairly insensitive to the choice of 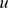 for values between 3 and 20.

**Figure 4:**
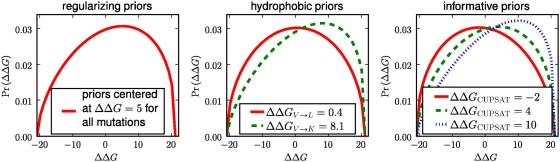
Prior distributions, 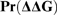, over the 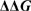 values. The “regularizing priors” are peaked at the moderately destabilizing value of 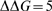 to capture the general knowledge that most mutations are destabilizing. The “hydrophobic priors” capture the knowledge that mutations that cause large changes in hydrophobicity are often more destabilizing. These priors are peaked at a value equal the the absolute value of the difference in amino acid hydrophobicity (as defined by the widely used Kyte-Doolittle scale [81]). For example, the prior for a mutation from hydrophobic valine (V) to similarly hydrophobic leucine (L) is peaked near zero, while that for mutation from valine to charged lysine (K) is peaked at a much more destabilizing value. The “informative priors” are peaked at the 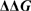 values predicted by the state-of-the-art physicochemically based program CUPSAT [8], and so are designed to leverage extensive pre-existing knowledge about 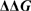 values. All the priors are fairly loose to make the 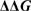 values responsive to their effect on the likelihood. The priors also help regularize [80] the 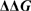 predictions by biasing them towards a reasonable range.

One of the strengths of our approach is that it allows for the use of informative priors 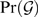 over the 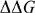 values. These priors can serve two purposes. One purpose is simply to prevent overfitting by regularizing [80] the 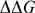 values by biasing them towards a central reasonable range. A second purpose is to actively incorporate some of the substantial existing knowledge about how protein structure and amino-acid character influence 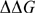 values. One piece of this knowledge is simply the general fact that most mutations to proteins are destabilizing, and so have 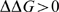. It is also known that mutations that cause large changes in the hydrophobicity of amino acids are often more destabilizing. At a more detailed level, there are a number of physicochemical modeling programs that attempt to make quantitative predictions of 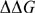 values from protein structural information [1]–[8]. We tested phylogenetic inference with priors incorporating information at all three of these levels, as shown in Figure 4. At the most basic level, we used “regularizing priors” that simply biased all the 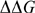 values towards the generally observed range of mildly to moderately destabilizing. A second set of “hydrophobic” priors were based on the idea that mutations that cause large changes in amino acid hydrophobicity will tend to be more destabilizing. For these priors, the prior estimate for each 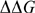 value was equal to the absolute value of the difference in the hydrophobicities of the wildtype and mutant amino acids, as given by the widely used Kyte-Doolittle hydrophobicity scale [81]. These hydrophobic priors therefore predicted that mutations that caused large changes in hydrophobicity would be highly destabilizing (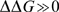), while those that led to small changes in hydrophobicity would have little effect on stability (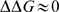). A third set of “informative priors” were designed to leverage the full available knowledge about the effects of mutations on stability. This knowledge is most completely encapsulated in various physicochemically-based prediction programs [1]–[8], which utilize a wide range of structural and biophysical information to make quantitative 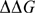 predictions for individual mutations. We chose one of these programs, CUPSAT [8], to predict 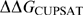 values for all single amino-acid mutations from the protein crystal structures. We chose the CUPSAT program because it has a publicly available webserver (http://cupsat.tu-bs.de) and has reported benchmarks that equal or exceed those of other prediction programs [8]. The prior estimate for each mutation was then the 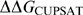 value predicted by CUPSAT, after rescaling the predictions as described below. For all three sets of priors, the prior 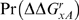 for mutating residue 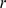 from A to 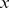 was a beta distribution probability density function peaked at the prior estimate for that mutation. The beta distribution functions were defined so that the sum of the alpha and beta parameters equaled three, and with the functions going to zero at the upper and lower limits of the allowed range for the 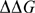 values. These prior functions are therefore broad, and loosely bias the 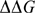 values toward the prior estimates. Examples of the priors are shown in Figure 4. The overall prior probability for the set 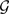 of 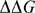 values was defined to the be product of the prior probabilities for the individual 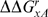 values, 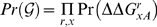.

In order for the phylogenetic inference to work effectively, it is necessary that the priors fall in the same numerical range over which the likelihood function is responsive to changes in the 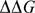 values. The actual 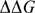 values of the phylogenetic inference approach have arbitrary units, so placing the priors in an appropriate dynamic range simply requires that the relevant parameters have compatible relative values. We set a 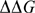 range of 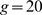, so that for all 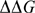 values, 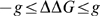. The values of the bin size 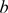 and the parameter 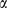 in Equation 3 are arbitrary, but serve to set the scale for how 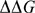 values affect the substitution probabilities. We chose a value of 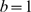, and a value of 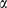 such that 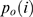 falls to one percent of its previous value every 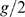 bins (this is 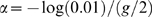). This scaling means that the substitution probabilities as a function of the 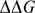 values can cover a large dynamic range of four orders of magnitude given the limits for the 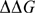 values set by 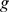. It is then necessary to choose priors that fall in the same dynamic range. For the regularizing priors, the prior estimate had a value of five for all 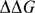 values, which corresponds to a moderately destabilizing mutation. For the hydrophobicity priors, we did not rescale the values obtained by taking the absolute value of the difference in Kyte-Doolittle hydrophobicities, since these values already fall in a reasonable range of zero to nine. For the informative priors, we rescaled the 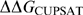 values to bring them into an appropriate range. Specifically, we rescaled them so that the difference between the values at the 10th and 90th percentiles was 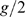 and the mean 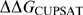 value was 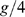, and truncated outlier values so that 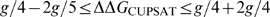.

Solving Equation 13 requires a numerical method for finding the value 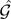 of 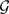 that maximizes the *a posteriori* probability. The 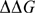 values for the different positions of the protein are independent, so we maximized the 19 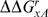 values for each position separately. For each residue 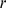, we first set the 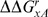 values to random numbers drawn from a normal distribution with a mean of zero and a standard deviation of 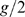. For each 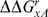, we then performed a line search to find the value that represented the nearest local maximum in the *a posteriori* probability. We repeated this procedure for the next 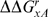 value, until we had performed line searches for all 19 values. This constituted one iteration of maximization of the 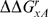 values; we continued performing iterations until no further local adjustments in any of the 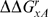 values increased the *a posteriori* probability. This maximization algorithm is stochastic, and we cannot guarantee that it converges to the global maximum (or indeed converges at all). However, in practice it always converged rapidly, and repeating the procedure with different random starting values led to highly similar 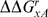 values at the completion of the maximization. We considered this ample evidence that this rather *ad hoc* algorithm was a sufficient method for solving the 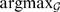 function of Equation 13. Implementing a more sophisticated gradient-based maximization is an area for future research, and may lead to improvements in computational speed. However, the PIPS program described above was sufficiently fast to be run on a laptop computer to give the predictions described in the next few sections.

### Comparison of phylogenetically inferred 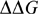 values with existing experimentally measured values for small soluble proteins

We first tested the phylogenetic inference approach on existing experimentally measured 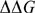 values. Most published 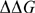 values are for mutations to a few small soluble proteins. We examined the ProTherm [82] database, and found that the proteins with the most 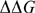 values were bacteriophage T4 lysozyme, sperm whale myoglobin, *Bacillus amyloliquefaciens* barnase, *Bacillus subtilis* cold shock protein, *Escherichia coli* ribonuclease HI, and *E. coli* thioredoxin. We then searched for sequences with least 50% identity to each of these six proteins in the UniRef100 database [83]. We found a substantial number of homologous sequences for cold shock protein (763 sequences), ribonuclease HI (239 sequences), and thioredoxin (213 sequences). We therefore chose these three proteins as the subjects of our analysis. For each protein, we extracted from the original references all available experimentally measured 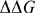 values for single amino acid substitutions, to obtain a total of 76 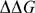 values for cold shock protein [84]–[89], 31 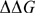 values for ribonuclease HI [90]–[96], and 32 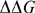 values for thioredoxin [14],[16],[97],[98].

In order to provide points of comparison, we first examined the ability of the physicochemical modeling program CUPSAT [8] and the consensus approach to predict the experimentally measured 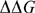 values for these three proteins. We used the CUPSAT webserver to predict 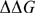 values from the protein crystal structures (PDB codes 1CSP [99] for cold shock protein, 2RN2 [100] for ribonuclease HI, and 2H6X for thioredoxin). We calculated the consensus approach predictions using the standard Boltzmann form where 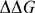 is the negative logarithm of the ratio of the frequencies of the mutant and wildtype residues in the alignment of homologous sequences (with a pseudocount of one added to the count for each amino acid before calculating the frequencies). We then used the PIPS program described in the previous section to make 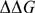 predictions by a phylogenetic inference approach. PIPS predictions were made using each of the three sets of priors (informative, regularizing, and hydrophobic) described in the previous section.

**Figure 5:**
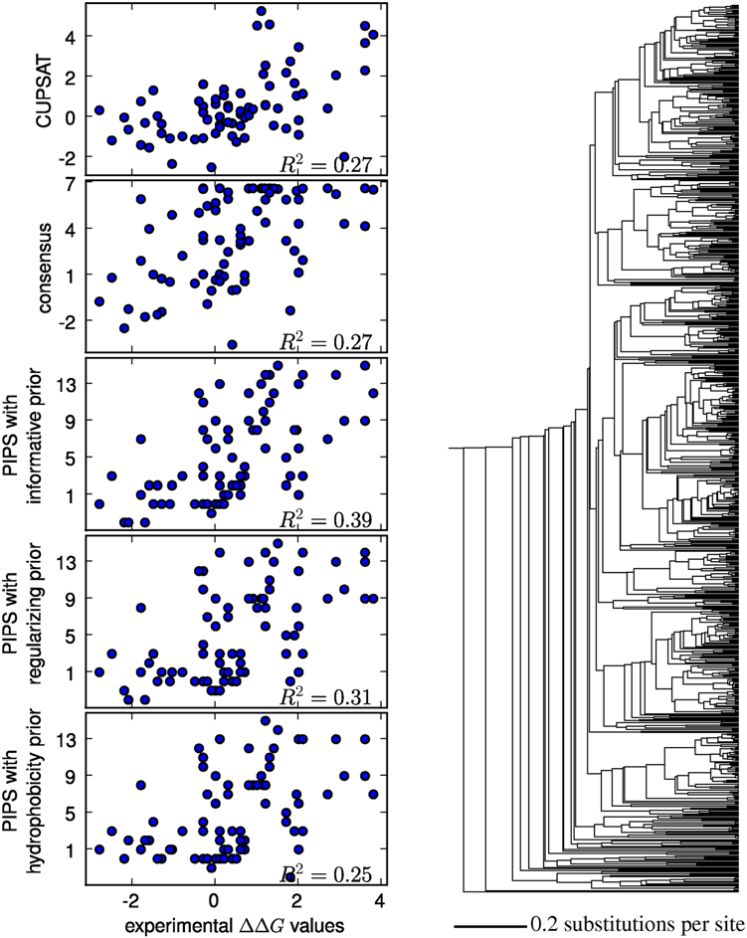
Experimentally measured and predicted 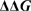 values for the 68-residue cold shock protein. The plots at left show the predictions made by the CUPSAT physicochemical modeling program, the consensus approach, and the PIPS phylogenetic inference program using the informative, regularizing, and hydrophobicity priors. To the right is the phylogenetic tree of 763 sequences that was utilized by the PIPS program. The 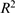 values are the squared Pearson correlation coefficients.

**Figure 6:**
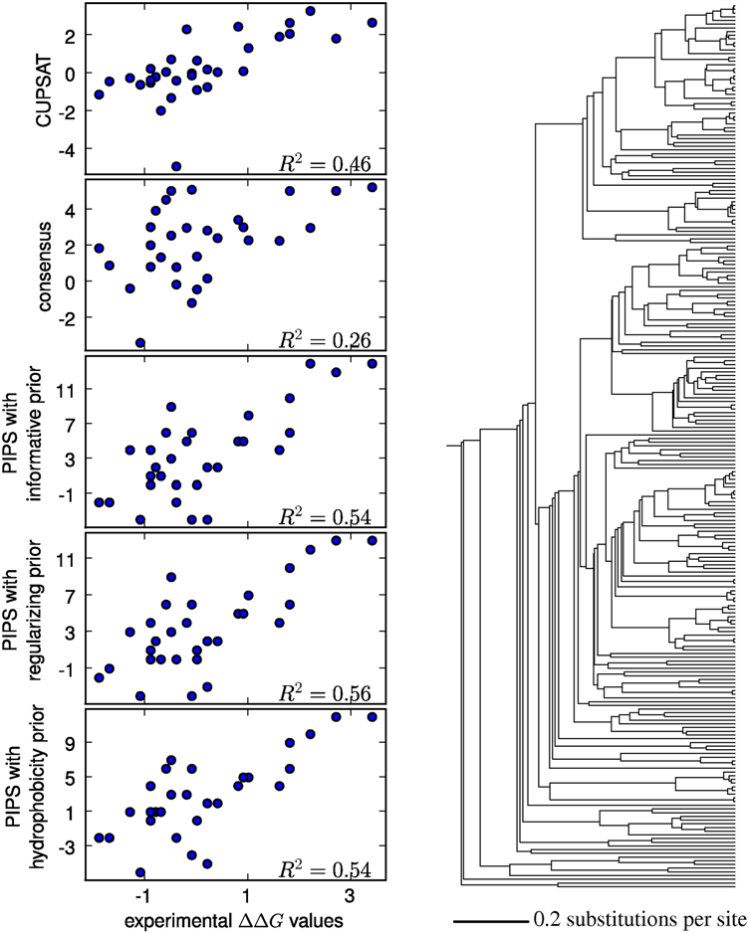
Experimentally measured and predicted 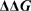 values for the 156-residue ribonuclease HI protein. The plots at left show the predictions made by the CUPSAT physicochemical modeling program, the consensus approach, and the PIPS phylogenetic inference program using the informative, regularizing, and hydrophobicity priors. To the right is the phylogenetic tree of 239 sequences that was utilized by the PIPS program. The 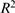 values are the squared Pearson correlation coefficients.

**Figure 7:**
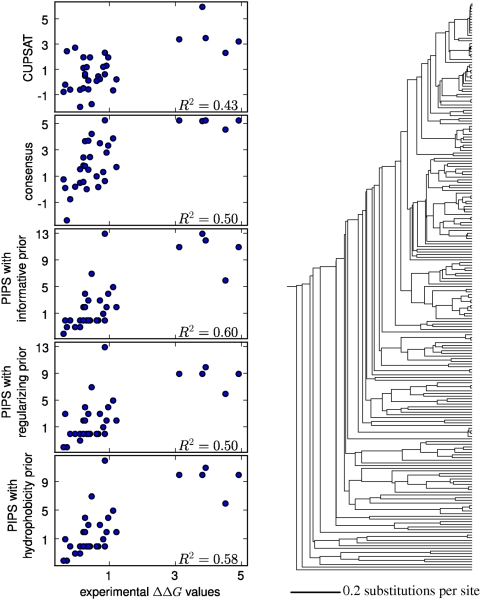
Experimentally measured and predicted 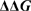 values for the 109-residue thioredoxin protein. The plots at left show the predictions made by the CUPSAT physicochemical modeling program, the consensus approach, and the PIPS phylogenetic inference program using the informative, regularizing, and hydrophobicity. To the right is the phylogenetic tree of 213 sequences that was utilized by the PIPS program. The 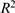 values are the squared Pearson correlation coefficients.

Figures 5, 6, and 7 show the correlations between the predicted and experimentally measured 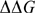 values for each of the three proteins. For each of the three proteins, all methods made predictions that were correlated with the experimental 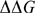 values (with 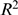 values ranging from 0.25 to 0.60), although there was also always substantial scatter in the correlation plots. In general, the PIPS program appeared to perform slightly better with the informative priors than with either the regularizing or hydrophobic priors. The PIPS program with the informative priors modestly but consistently outperformed both CUPSAT and the consensus approach (with the 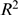 values for the PIPS program exceeding those for CUPSAT and the consensus approach by amounts ranging from 20% to two-fold). Because these correlations are with experimental data spanning a wide range of stabilizing and destabilizing 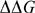 values, it is difficult to discern whether PIPS is also clearly better at identifying the most stabilizing mutations (the metric that would be most relevant for engineering protein stability), although it perfoms at least as well as consensus and CUPSAT in this respect. In any case, we interpret the higher overall correlations obtained with PIPS to indicate that for small soluble proteins, the phylogenetic inference approach with informative priors is more accurate than both a state-of-the-art physicochemical modeling program and the consensus approach. In the remainder of this work, all PIPS predictions are made with the informative priors.

**Figure 8:**
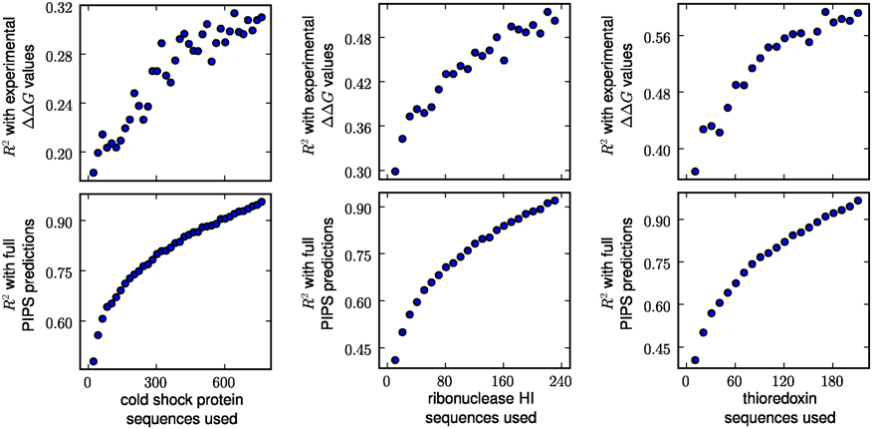
Performance of the phylogenetic inference approach as a function of the number of sequences used. The PIPS predictions using informative priors were run using subsets of all of the available protein sequences. The resulting 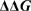 predictions were then correlated with the experimental 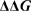 values (top) or the PIPS 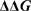 predictions obtained using all available sequences (bottom). The 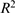 values are the squared Pearson correlation coefficients. For each number of sequences used, the PIPS predictions were made using 10 different random sequence subsets, and the displayed 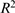 values are the average correlations over these 10 subsets. For cold shock protein, the subsets were made at intervals of 20 sequences, while for ribonuclease HI and thioredoxin they were made at intervals of 10 sequences.

The phylogenetic inference approach determines 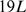 different 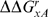 values for each protein, where 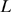 is the length of the protein. Because such a large number of parameters is being inferred, it is interesting to examine how the performance of the phylogenetic inference depends on the number of sequences used. One way to do this is to make PIPS predictions using a random subset of all of the available sequences, and then to correlate these predictions with the experimentally measured 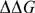 values, or with the PIPS predictions made using all available sequences. We performed such an analysis for all three proteins. Figure 8 shows the results of this analysis. Not surprisingly, using larger numbers of sequences improves the accuracy of the predictions, as measured by the correlations with both the experimental 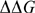 values and those predicted by PIPS using all available sequences. However, the correlations are still quite good when only a fraction of the available sequences are used. These results indicate that although it is obviously advantageous to use more sequences, phylogenetic inference performs fairly well even if only 50 or 100 sequences are used. We suggest that both the informative and regularizing [80] aspects of the Bayesian priors serve to prevent overfitting and guarantee reasonable predictions even when the number of sequences is small.

### Test of phylogenetic inference approach's ability to identify known temperature-sensitive and revertant mutations to influenza hemagglutinin

We next tested the phylogenetic inference approach on the more difficult problem of identifying stabilizing mutations to influenza hemagglutinin. Hemagglutinin is a 565-residue trimeric membrane-bound glycoprotein that mediates the binding and fusion of influenza virus with target cells, making it much larger and more complex than most proteins that have been successfully modeled using physicochemical approaches. Influenza has been the subject of intensive sequencing efforts, and so a large number of hemagglutinin sequences are available in the publicly accessible Influenza Virus Resource [101] (http://www.ncbi.nlm.nih.gov/genomes/FLU/FLU.html). However, these sequences contain unusual patterns of phylogenetic relationship, due to the distinctive selection pressures operating on influenza in humans [102] and birds [103], as well as the fact that most sequencing has focused on a few subtypes of special interest (such as avian H5N1 and human H3N2 and H1N1 viruses). The complexity of the hemagglutinin protein and the strong evolutionary relationships among the available sequences are likely to make the prediction of stabilizing mutations a challenging problem for any method.

As an test data set, we used a collection of previously described mutants to the hemagglutinin of the A/WSN/33 (H1N1) influenza virus. This set contains two temperature-sensitive virus mutants that can replicate only at reduced temperatures (34°C but not 39.5°C) due to a failure of the hemagglutinin protein to be transported to the cell membrane at elevated temperatures [104]. The hemagglutinin proteins of these temperature-sensitive viruses also show an increased loss of hemagglutination activity at high temperature, suggesting general defects in both folding and stability [104]. Each of the two temperature-sensitive viruses is defective due to a different single amino-acid mutation in hemagglutinin [105]. These two temperature-sensitive mutations constitute our set of “destabilizing” mutations. For one of the two temperature-sensitive mutants (the one designated as ts-134 in [104]–[106]), a collection of second-site revertant mutations in hemagglutinin have been isolated by selecting for viruses that have regained the ability to grow at elevated temperatures [105],[106]. These revertant mutations presumably enhance hemagglutinin's folding and/or stability. There are 16 different revertant mutations, which constitute our set of “stabilizing” mutations.

**Figure 9:**
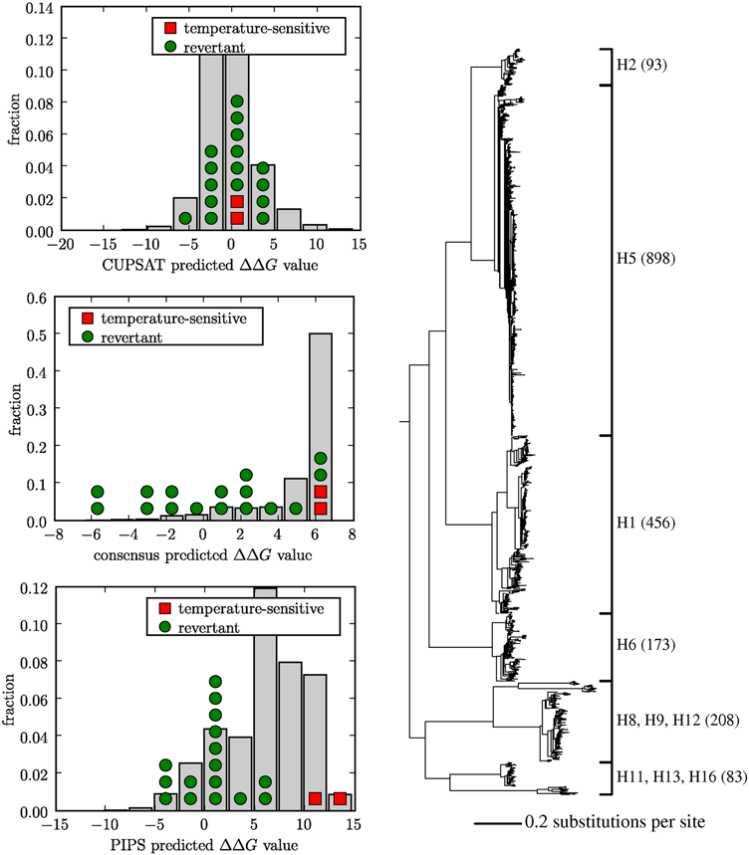
Predicted stability effects of known temperature-sensitive and revertant mutations to H1 hemagglutinin. In the plots at left, bars indicate the distribution of predicted 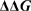 values for all single mutations, while symbols show predicted values for the temperature-sensitive and revertant mutations. At right is the phylogenetic tree utilized by the PIPS program. The tree labels give the hemagglutinin subtypes and corresponding numbers of sequences. The PIPS predictions are made using the informative priors.

We tested the ability of the CUPSAT program, the consensus approach, and the PIPS program (using the informative priors) to distinguish the temperature-sensitive and revertant mutations. The CUPSAT predictions were made using the crystal structure of the A/PR/8/34 (H1N1) hemagglutinin (PDB code 1RVZ [107]), which is closely related to the A/WSN/33 (H1N1) hemagglutinin (90% sequence identity over the 487-residue portion of the protein present in the crystal structure). For sequence data, we used the full-length hemagglutinin sequences (lab strains excluded) present in the Influenza Virus Resource [101] at the time of our initial analysis (September, 2007). We made no restriction on the host species of the virus, since we assume that the basic requirements for protein folding and stability should be similar in all hosts. We restricted our analysis to those hemagglutinin subtypes with at least close to 50% protein sequence identity to H1 hemagglutinins (sequences from the H1, H2, H5, H6, H8, H9, H11, H12, H13, and H16 subtypes) and excluded sequences from more distantly related subtypes (H3, H4, H7, H10, H14, and H15). This yielded 1,911 unique hemagglutinin sequences, which were used to build the phylogenetic tree shown in Figure 9.

Figure 9 shows the predicted stability effects of the temperature-sensitive and revertant mutations from each of the three methods. The CUPSAT program had no ability to distinguish the temperature-sensitive and revertant mutations, since it predicted the stability effects of all of these mutations to be clustered together near the center of the distribution of effects for all mutations. This suggests that either hemagglutinin is too large or mobile for effective physicochemical modeling, or that the CUPSAT program is overfit on the set of small soluble proteins on which it was parameterized (which includes cold shock protein, ribonuclease HI, and thioredoxin). The consensus approach could partially distinguish the temperature-sensitive and revertant mutations, predicting most of the revertant mutations to be more stabilizing than the temperature-sensitive mutations. However, the PIPS program was clearly the most successful approach, cleanly predicting that all of the revertant mutations should be more stabilizing than both of the temperature-sensitive mutations. These results support the findings of the previous section that the PIPS program is more accurate than either the physicochemical modeling program or the consensus approach, and suggest that the extent of its superiority over physicochemical modeling is greater for more complex proteins such as hemagglutinin.

### Prediction and experimental verification of new stabilizing mutations to influenza hemagglutinin

We next tested whether the phylogenetic inference approach could predict entirely new stabilizing mutations to influenza hemagglutinin. Our experimental strategy for performing this test was to introduce stabilizing mutations predicted by PIPS into A/WSN/33 (H1N1) hemagglutinin carrying a known temperature-sensitive mutation (the single mutation responsible for the ts-134 phenotype [105]) and examine whether these predicted stabilizing mutations actually allowed the virus to grow at elevated temperatures.

**Figure 10:**
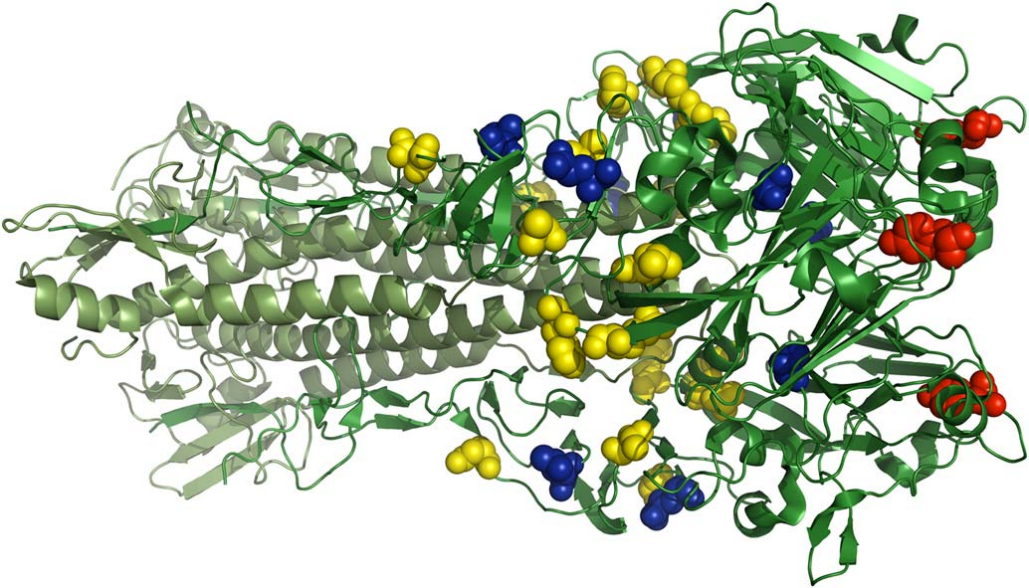
Locations of the predicted and confirmed stabilizing mutations to H1 hemagglutinin. The full hemagglutinin trimer is shown in green, with the HA1 chains in dark green and the HA2 chains in light green. The temperature-sensitive mutation (ts-134 [104]–[106]) is shown with red spheres. The yellow spheres show the mutations that were predicted to be stabilizing by the PIPS program. The blue spheres show the four predicted mutations that were experimentally confirmed to actually increase the temperature stability. The structure is PDB code 1RVZ [107].

The PIPS analysis described in the previous section identified 23 different mutations to A/WSN/33 H1 hemagglutinin that were predicted to be the most highly stabilizing (these are the mutations with PIPS 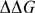 values less than −5 that appear in the small left-most bar of the histogram in Figure 9). Seven of these mutations are to residues in the antigenic sites of H1 hemagglutinin (as delineated in [108]), and so are likely subject to positive selection for diversification. Since one of the basic assumptions of the phylogenetic inference approach is that mutations are selected only for their effects protein stability, we excluded these seven mutations. Another mutation occurs in the N-terminal signal sequence, and so was excluded since it is not present in the final folded structure. Three of the mutations occured in the HA2 polypeptide; we excluded these three mutations since the temperature-sensitive mutation is found in the HA1 polypeptide. This left 12 predicted stabilizing mutations in the HA1 polypeptide. The locations of these predicted stabilizing mutations in the three-dimensional structure are shown in Figure 10. None of these mutations is among the known revertants [106] described in the previous section.

We introduced these 12 predicted stabilizing mutations into the hemagglutinin gene on the background of the temperature-sensitive mutation using site-directed mutagenesis. We then created the mutant viruses at 34.0°C using the influenza reverse genetics system [109], as described in more detail in the *Methods* section. The viruses were then plaqued on confluent Madin Darby canine kidney (MDCK) cells at 34.0°C, 35.5°C, 37.0°C, 38.5°C, and (in most cases) 40.0°C.

Table 1 summarizes the results of these plaque assays. The wildtype virus plaqued at all five temperatures, with some reduction in plaque size and clarity at 40°C. The virus carrying the temperature-sensitive mutation in hemagglutinin plaqued at 34.0°C and 35.5°C, formed smaller and more opaque plaques at 37.0°C, and formed no visible plaques at 38.5°C and 40.0°C. Of the 12 mutant viruses, one failed to express in the reverse genetics system. Three appeared to be slightly less stable than the temperature-sensitive parent virus, plaquing only at 34.0°C and 35.5°C. Four had similar profiles to their parent virus, plaquing well at 35.5°C but only weakly at 37.0°C. The other four mutant viruses exhibited clearly enhanced thermotolerance, plaquing well at 37.0°C and weakly at 38.5°C.

**Table 1:** Plaque growth of influenza A/WSN/33 (H1N1) viruses carrying mutations in hemagglutinin.

Mutant	34.0°C	35.5°C	37.0°C	38.5°C	40.0°C
WT	++	++	++	++	+
ts	++	++	+	−	−
ts-D51K (D39K)	+	+	−	−	ND
ts-D51R (D39R)	+	+	−	−	ND
ts-A64K (A52K)	++	++	+/−	−	ND
ts-Q67I (Q55I)	++	++	++	+/−	−
ts-D110E (D98E)	++	++	++	+/−	−
ts-L121F (L109F)	+	+	+/−	−	−
ts-R274K (R262K)	+	+	−	−	ND
ts-R274Q (R262Q)	−	−	−	−	ND
ts-F276G (F264G)	++	++	+/−	−	−
ts-T282Q (T270Q)	++	++	+	−	−
ts-Q298K (Q286K)	++	++	++	+/−	−
ts-Q298R (Q286R)	++	++	++	+/−	−

Results are for wildtype (WT), temperature-sensitive (ts), and ts virus with predicted stabilizing mutations. The plaques are scored as ++ for clear plaques, + for smaller or opaque plaques, +/− for barely distinguishable plaques, − for no plaques, and ND for not determined. The first mutation numbers are for sequential numbering of the A/WSN/33 hemagglutinin sequence beginning with zero at the N-terminal methionine, while the numbers in parentheses correspond to those used in the crystal structure with PDB code 1RVZ.

**Figure 11:**
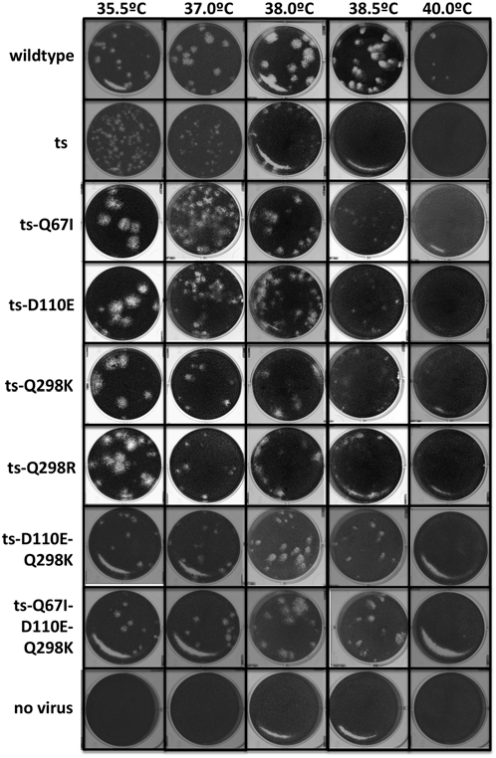
Plaque assays of wildtype, temperature-sensitive (ts), and ts influenza with predicted stabilizing hemagglutinin mutations. All four of the single mutations allow the virus to plaque at higher temperatures than the ts parent. The multiple mutants plaque more effectively at higher temperatures than the single mutants. Mutations are named according to the numbering scheme described in Table 1.

To confirm the increased temperature stability of viruses carrying the four apparently stabilizing mutations, we re-grew the viruses from the encoding plasmids and again plaqued them at various temperatures that now included 38.0°C. The results of these plaque assays are shown in Figure 11. All four mutants were clearly more thermotolerant than their temperature-sensitive parent, although still less so than the wildtype virus. To test whether the stabilizing mutations had cumulative effects, we constructed a double-mutant carrying two of the stabilizing mutations, and a triple-mutant carrying three of the stabilizing mutations. As can be seen in Figure 11, these multiple mutants were more thermotolerant than the single mutants, as indicated by better plaquing at 38.5°C.

## Discussion

The most compelling evidence for the essential validity of the phylogenetic inference approach presented here is also the source of its greatest potential utility — the fact that it is able to predict experimentally measured mutational effects on stability. We found that it predicted known 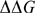 values for single amino acid mutations to small soluble proteins with an accuracy exceeding that of either of two existing strategies, the consensus approach or a state-of-the-art physicochemical modeling program. Phylogenetic inference also was able to distinguish between known temperature-sensitive and revertant mutations to influenza hemagglutinin, a large multimeric protein that evolves under distinctive selection pressures. The extent to which phylogenetic inference outperformed the consensus approach and especially physicochemical modeling was greater for hemagglutinin than for the small soluble proteins, suggesting that it may be most useful on precisely the more complex proteins that are often of greatest interest in biology and biomolecular engineering.

Our most stringent test of the phylogenetic inference approach was to use it to predict new mutations to hemagglutinin that rescued the growth of a temperature-sensitive influenza virus. Of the 12 predicted stabilizing mutations, four were indeed detectably stabilizing, four had little effect, three were slightly destabilizing, and one appeared to be lethal. How good (or bad) was this performance? Because we did not experimentally test CUPSAT and consensus predictions of stabilizing mutations to hemagglutinin, we cannot directly compare these two methods to phylogenetic inference in this respect. Comparison of the three methods on the set of previously known stabilizing mutations to hemagglutinin (Figure 9) strongly suggests that CUPSAT is unable to reliably distinguish stabilizing and destabilizing mutations to hemagglutinin, but only weakly suggests that phylogenetic inference is superior to the consensus approach in this regard. It therefore remains possible that the consensus approach would have made equally successful predictions. Another benchmark of the phylogenetic inference approach's predictions would be a comparison with a set of random single amino acid mutations to hemagglutinin. But the extensive amount of work required to generate and characterize such a panel of random mutants dissuaded us from carrying out such an experiment. Others seem to have been similarly dissuaded, since we are unaware of any published analyses of the stability effects of truly random mutations even to more experimentally tractable proteins. However, there have been coarse-grained analyses in the form of protein engineering experiments that screen for random mutations that enhance stability. Such experiments typically isolate one detectably stabilizing mutation for every 300 to 1,000 screened (frequencies of 0.4% for an esterase [110], 0.1% for subtilisin [111], 0.1% for a haloalkane dehalogenase [112], 0.2% for a phytase [113], and 0.1% for a fructosyl-amino acid oxidase [114], although methodologies vary widely). Assuming these frequencies can be extrapolated to hemagglutinin, the phylogenetic inference approach's success rate of four in 12 represents an improvement of two to three orders of magnitude over the random expectation — although of course two-thirds of the predicted stabilizing mutations still failed to enhance the virus's thermotolerance. Given these results, as well as the improved but still imperfect predictions of known 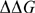 values, we can simultaneously ask both why the phylogenetic inference approach performs so well and why it does not perform better.

The phylogenetic inference approach performs so well because it ties protein stability to the underlying selection pressures, and so can draw from the full evolutionary histories of homologous proteins. Existing sequence-based strategies such as the consensus approach only consider the final evolved sequences, and so may miss some of the information contained in the substitution probabilities implied by the protein phylogeny. Physicochemical modeling utilizes knowledge about the biophysical forces that determine a protein's structure. But analyzing mutations with physicochemical modeling is more difficult than simply scoring the relative energies of different conformations of the same sequence, since a mutation can induce a change in the unfolded state. Computational descriptions of the unfolded state are still in their infancy, so it may be a long time until physicochemical modeling incorporates all of the subtleties needed to make fully accurate predictions. However, the phylogenetic inference approach leverages the incomplete but substantial knowledge already encapsulated by physicochemical modeling to build informative priors. These priors serve as reasonable initial guesses for the mutational effects on stability, which are then improved based on the substitution probabilities extracted from the protein phylogenies. Both the power of physicochemical modeling and the number of available protein sequences are likely to continue to increase, and as they do, the accuracy of the phylogenetic inference approach should improve correspondingly.

Why does the phylogenetic inference approach not perform better? The approach involves a number of mathematical and conceptual approximations. We are inclined to believe that the most limiting is the idea that all selection on amino acid substitutions occurs along the single additive dimension of protein stability. Clearly this assumption is inaccurate for the (probably small [36]–[39]) fraction of residues specifically involved in protein function. But it is also imperfect for the much larger fraction of residues with no direct functional role. These residues are constrained by selection on properties in addition to stability, including folding efficiency [44], kinetic stability [16],[45], and resistance to aggregation [40]–[43]. Furthermore, even the biologically relevant measure of stability is somewhat unclear. The study of protein stability was pioneered [115] on small proteins that fold reversibly *in vitro*, allowing for true thermodynamic measurements of 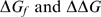 values [53]. However, many proteins do not fold reversibly [45],[54],[116], and even for those that do, the measured stabilities can be sensitive to the solvent conditions [89],[117],[118], which are usually quite different from the *in vivo* cellular milieu [119]. The saving grace from these complications is that different measures of protein stability (thermodynamic, thermal, chemical, proteolytic, kinetic) are substantially correlated with each other [16],[55],[61],[120], and to a lesser degree with folding efficiency [46]–[49] and resistance to aggregation [40]. The phylogenetic inference approach works to the extent that all of these properties can be grouped under the generalized concept of protein stability, and fails to the extent that mutations have distinct effects on each of them. So the inability of some of the predicted stabilizing mutations to rescue influenza's thermotolerance simply means that they did not compensate the original hemagglutinin defect (poor transport from the Golgi and decreased resistance to thermal inactivation [104])—they may still benefit related properties that were not compromised in this particular virus. Ultimately, such issues can be addressed only by relating the full spectrum of a mutation's biophysical effects to its tendency to be fixed by evolution, a type of analysis that should also help resolve the hotly debated question of what selection pressures account for observed patterns of protein evolution [121],[122].

Despite these issues, the approach presented here is a clear conceptual improvement over the traditional concept of matrices specifying fixed “average” amino acid substitution tendencies that are unrelated to on any specific experimental measurement. Even recent work [19]–[28] that uses sophisticated simulations or structural analysis to derive site-specific substitution matrices ultimately fails to connect the substitution tendencies along protein phylogenies to any experimentally tangible properties of the mutations. By making such a connection, our approach reverses the usual tactic of maximum likelihood and Bayesian phylogenetic tree reconstruction. In those methods, some amino acid substitution model is assumed, and then used to infer a phylogenetic tree. Here we have assumed the phylogenetic tree, and then used it to infer the effects of individual mutations on stability. Ultimately, it would be most satisfactory to infer both the phylogenetic tree and the stability effects directly from the protein sequences, perhaps with the assistance of informative priors derived from physicochemical modeling. Performing such a dual inference would of course raise daunting computational issues of adequately sampling from the distributions of both possible tree topologies and mutational effects. However, progress in such a direction could ultimately lead to strategies for analyzing homologous sequences that yield useful information about both evolutionary histories and protein biophysics.

## Methods

### Cloning of plasmids

The eight bidirectional polymerase I/polymerase II influenza reverse genetics plasmids [109] for the A/WSN/33 (H1N1) strain (pHW181-PB2, pHW182-PB1, pHW183-PA, pHW184-HA, pHW185-NP, pHW186-NA, pHW187-M, and pHW188-NS) as well as the null cloning plasmid (pHW2000) were kind gifts from Robert G. Webster at St. Jude Children's Research Hospital. The plasmid pHW184-HA-ts134 was constructed by introducing the single mutation responsible for the ts-134 temperature-sensitive phenotype [105] (Y173H in the numbering scheme where the N-terminal methionine is zero) into hemagglutinin by strand overlap extension PCR, and cloning the insert into the BsmBI restriction sites of pHW2000. A similar procedure was then used to individually construct the 12 predicted stabilizing mutations shown in Table 1 on the background of this temperature-sensitive mutation to yield the plasmids pHW184-ts134-D51K, pHW184-ts134-D51R, *etc.* The accuracy of all plasmids was confirmed by sequencing the hemagglutinin genes and immediate flanking sequences.

### Cells and media

The 293T human embryonic kidney cell line and the Madin-Darby canine kidney (MDCK) cell line were initially purchased from ATCC (CRL-11268 and CCL-34, respectively). The cells were maintained in D10 media, consisting of Dulbeccos's Modification of Eagle's Medium (DMEM, Cellgro 10-013-CV) supplemented with 10% heat-inactivated fetal bovine serum (HI FBS, Omega Scientific FB-01), 2 mM L-glutamine (Cellgro 25-005-CI), and 100 U/ml penicillin and 100 µg/ml streptomcyin (P/S, Bio-Whittaker 17-602E). Cells were passaged using 0.25% trypsin/2.21 mM EDTA when they reached 90–100% confluence, and were restarted from frozen stocks stored in liquid nitrogen roughly every month. All cells were maintained at 37°C with 5% carbon dioxide, except when the temperature was changed as indicated.

During influenza infections, cells were maintained in influenza growth medium with trypsin (IGM+T), consisting of OptiMEM I (Gibco 31985) supplemented with 0.01% HI FBS, 0.3% bovine serum albumin (BSA, Invitrogen 15260-37), P/S, 100 µg/ml calcium chloride, and 2 µg/ml of tosyl-phenylalanyl-chloromethyl-ketone (TPCK)-treated trypsin (Sigma Aldrich T-8802) [123]. For plaque assays, 2X IGM+T was prepared from OptiMEM I powder packets (Gibco 22600-050) with 2.4 g of sodium bicarbonate per packet in addition to 2X concentrations of the other components of IGM+T. The TPCK-trypsin was always added fresh immediately before use.

### Influenza reverse genetics

The influenza virus was reconstituted from the eight bidirectional reverse genetics plasmids [109] by co-transfecting 250 ng of each plasmid into a co-culture of MDCK and 293T cells in a 6-well plate. The co-cultures were seeded the day before with 5×10^5^ 293T and 3×10^5^ MDCK cells so that the plates were 50–80% confluent at the time of transfection. All transfections were performed using Mirus Transit293 transfection reagent. Post-transfection, plates were maintained at 34 m°C in order to allow growth of temperature-sensitive viruses. At 12–18 hours post-transfection, the media was changed to IGM+T (with two washes with phosphate buffered saline, PBS). After 24 hours of growth in IGM+T, 500 µl of the supernatant was passaged to fully confluent MDCK cells in IGM+T to expand the virus. The supernatant from the passage plate was collected after an additional 24–48 hours of growth, at which point significant virus-induced cell cytopathic effects were typically observed. The virus-containing supernatant was passed through a 0.45 µm filter, aliquoted, and stored at −80°C. All experiments involving influenza virus were performed in accordance with Biosafety Level 2 containment procedures.

### Plaque assays

For viral plaque assays, 6-well plates were seeded with 3.5×10^5^ MDCK cells per well so that they reached full confluence in 48 hours. Frozen aliquots of virus were thawed and serial 10-fold dilutions of virus were made in IGM+T. The confluent MDCK cells were washed twice with PBS, and then inoculated with 700 µl of the appropriate virus dilution. The 6-well plates were then transferred to a tissue culture incubator set at the appropriate temperature for 45 minutes, with occasional gentle tilting of the plate to spread the inoculum. An overlay medium was prepared by mixing equal volumes of 2X IGM+T and a 2.4% Avicel microcrystalline cellulose (FMC Biopolymer RC-581) suspension [124]. After the 45 minute incubation, 3 ml of overlay was added to each well and the plates were grown at the appropriate temperature undisturbed for 72 hours. The overlay was then removed by aspiration and the residual Avicel was removed by washing twice with PBS. The cell layer was stained by a 10–20 minute incubation with 0.1% crystal violet in 20% ethanol. The stain was removed with two additional PBS washes, and the plaques were photographed using a gel imager to yield photos like those shown in Figure 11.

Every effort was made to perform the plaque assays consistently, but there was still moderate variation in plaque size, number, and morphology when virus from the same stock was independently plaqued on different days (possibly due to slight variations in the conditions of MDCK cells). Because of the large amount of labor involved, it was of course impossible to perform all of the plaque assays on the same day. Figure 11 shows representative results, but some of the variation in plaque size and morphology may still be due to day-to-day variation. However, all mutants shown in Figure 11 were plaqued in independent experiments on different days using different initial viral stocks, and presence/absence of plaques at the different temperatures was repeatable, despite the modest variations in plaque morphology As can be seen in Figure 11, a crescent-shaped patch sometimes appeared in the lower-left corner of the MDCK monolayer. This patch occasionally appeared even in the absence of virus, and is probably due to cell drying or death rather than viral growth.
